# Supplementary material for: Transcriptome-wide analysis of the function of Ded1 in translation preinitiation complex assembly in a reconstituted in vitro system
Source: bioRxiv. 2024 Feb 5:2023.10.16.562452. Originally published 2023 Oct 16. Preprint. [Version 2] doi: 10.1101/2023.10.16.562452 (PMC10659408; doi:10.1101/2023.10.16.562452)
Supplement: Supplement 3 — Figure 1-S1. A schematic diagram of mRNA recruitment in translation initiation. The capbinding protein complex eIF4F, consisting of eIF4E, eIF4G and eIF4A subunits attaches to the cap at the mRNA 5’ end. The 43S PIC, which includes the 40S ribosomal subunit, eIFs 1, 1A, 3 and 5 and the eIF2•GTP•Met-tRNAi ternary complex, binds to the 5’ end of mRNA with the aid of the eIF4F complex and eIF4B and scans downstream in search of the start codon. A stable 48S-PIC is formed when a start codon is recognized. Figure 1-S2. Internal spike-in normalization controls using preformed 48S PICs on non-native mRNAs. (A) Steps in applying 48S-PIC spike-in controls for data normalization. Firefly and Renilla luciferase mRNAs (FLUC and RLUC) were in vitro transcribed from PCR-amplified gene templates containing T7 promoters and enzymatically capped using previously described protocols (Acker et al. 2007). The 48S PICs formed on the spike-in mRNAs were prepared separately from the testing samples in a recruitment reaction followed by RNase I treatment to digest unprotected mRNA. A constant amount of spike-in 48S PICs were resolved by sedimentation through the same sucrose gradients containing the experimental samples. The RPFs from these spike-in PICs served as internal controls for losses occurring during library construction and allowed data normalization across experimental samples. For deep sequencing data processing, non-coding RNA reads were removed and the remaining RPFs were aligned to spike-in mRNA sequences for calculating sample size factors and also aligned to the yeast genome to produce RPF counts on all yeast mRNAs. The RPF counts for all mRNAs in the genome were applied to DESeq2 using sample size factors calculated from the geometric means of spike-in mRNA RPFs mapped to FLUC/RLUC main AUGs and a number of internal AUGs. Recruitment efficiency (RE) values were calculated by normalizing the size-factor corrected RPFs to input mRNA densities. (B-D) Scatterplots of FLUC and [file NIHPP2023.10.16.562452v2-supplement-3.pdf]

bioRxiv preprint doi: https://doi.org/10.1101/2023.10.16.562452; this version posted February 5, 2024. The copyright holder for this preprint (which was not certified by peer review) is the author/funder. This article is a US Government work. It is not subject to copyright under 17 USC 105 and is also made available for use under a CC0 license.

Table S1: Oligonucleotides used for Spike-in mRNA template amplification

Primer name	Sequence (5' – 3')
T7-FLUC	AAGGAATTCATCTTAACTTT**TAATACGACTCACTATAG**GGCAAAC AAACAAACCAAAACCAAAACCACA***ATG***GAAGACGCCAAAAACATA
T7-RLUC	AAGGAATTCATCTTAACTTT**TAATACGACTCACTATAG**GGCAAAC AAACAAACCAAAACCAAAACCACC***ATG***ACTTGCAAAGTTTATGAT
FLUC-R	GTCGACGAGGAATTCATTATCAGTGC
RLUC-R	GTCGACTTCTCCTTCTTCAGATTTGATC

Note: T7 promoter sequences are in boldface and underlined; mAUGs are in boldface and italicized

Figure 1-S1

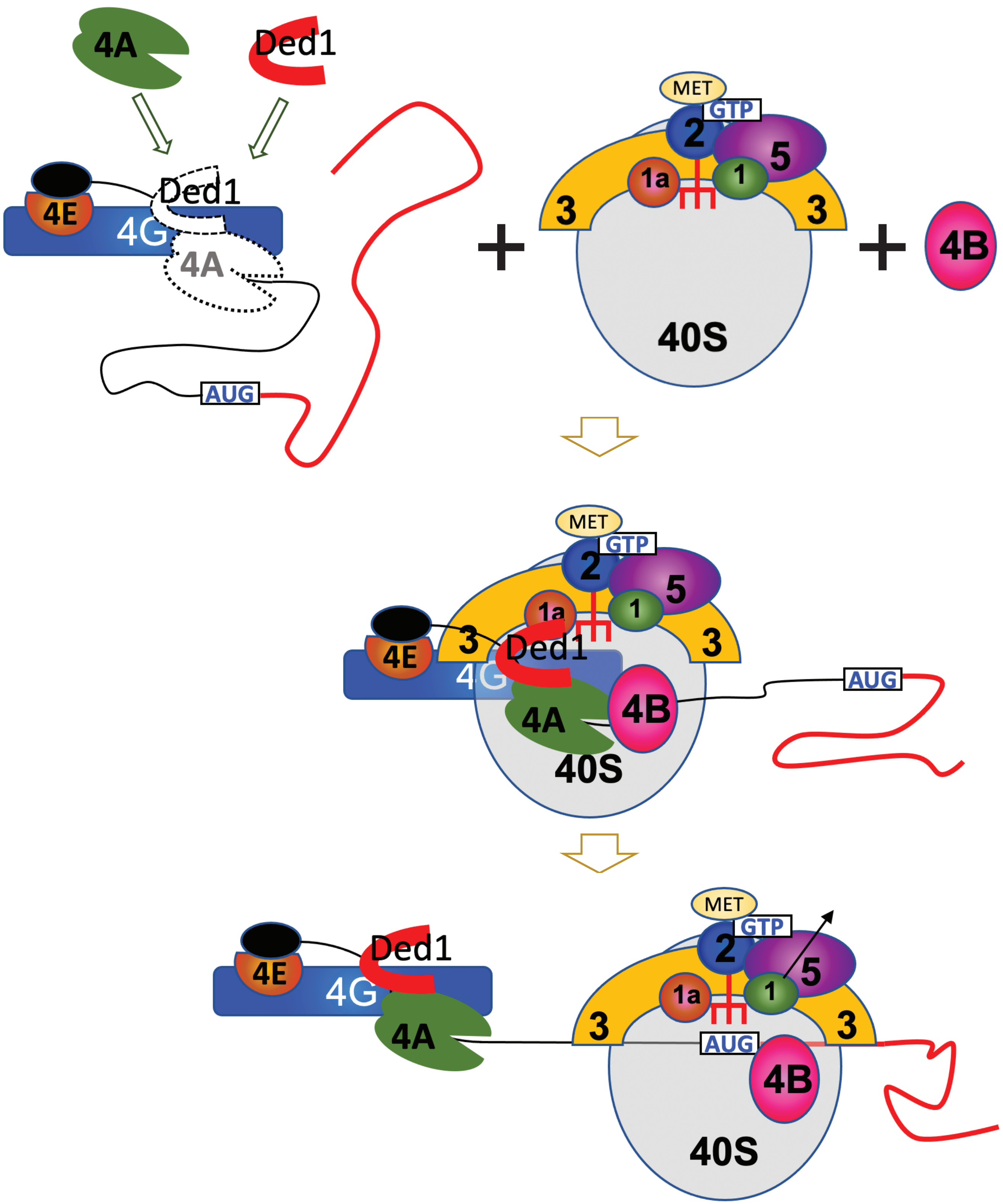
4A
Ded1
4E
4G
Ded1
4A
AUG
MET
GTP
2
5
1a
1
3
3
40S
4B
MET
GTP
2
5
1a
1
3
3
4E
Ded1
4G
4A
4B
40S
AUG
MET
GTP
2
5
1a
1
3
3
4E
Ded1
4G
4A
AUG
4B
40S

Figure 1-S2

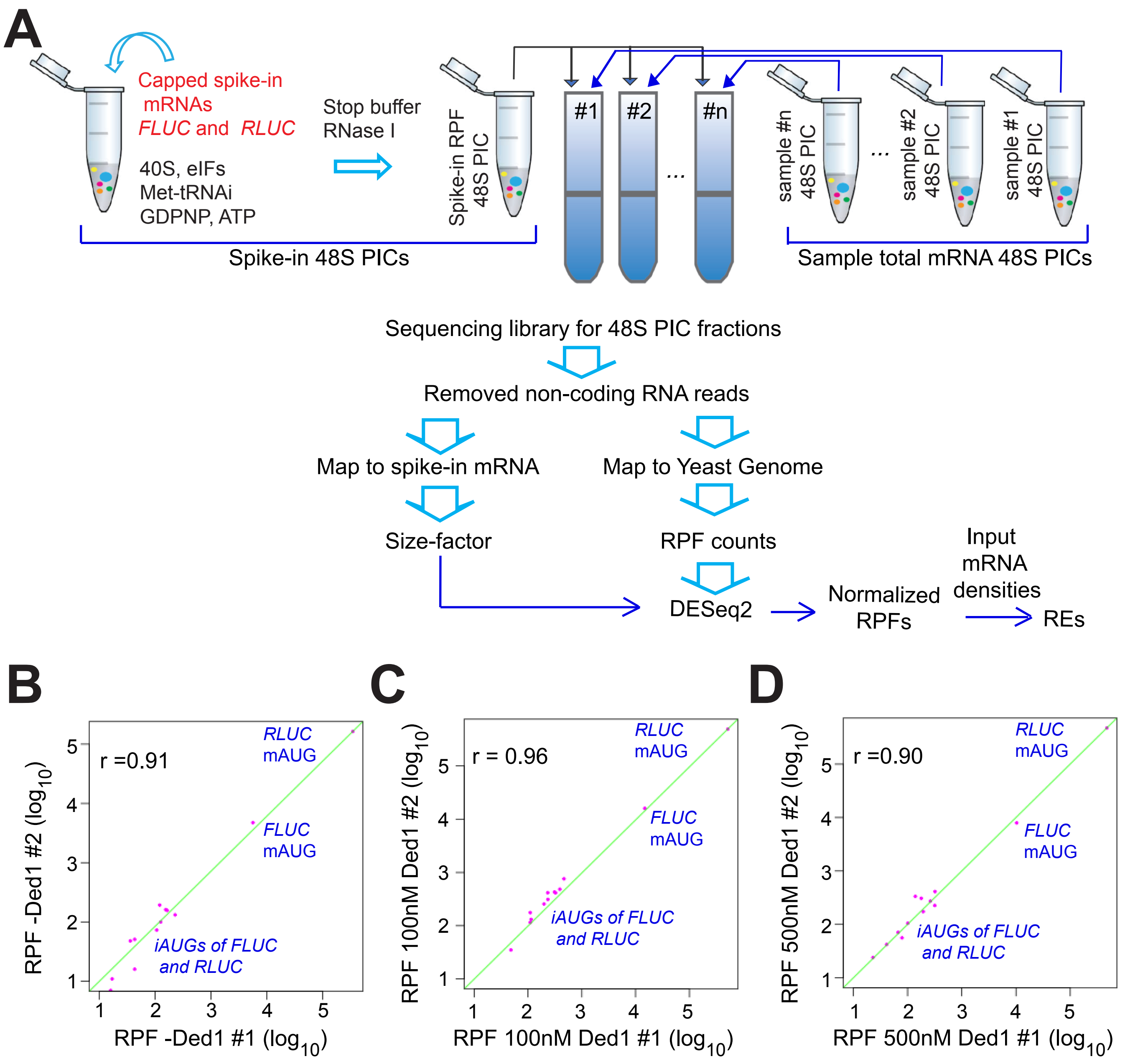
A
Capped spike-in mRNAs
FLUC and RLUC
40S, eIFs
Met-tRNAi
GDPNP, ATP
Stop buffer
RNase I
Spike-in RPF 48S PIC
#1
#2
...
#n
sample #n 48S PIC
...
sample #2 48S PIC
sample #1 48S PIC
Spike-in 48S PICs
Sample total mRNA 48S PICs
Sequencing library for 48S PIC fractions
Removed non-coding RNA reads
Map to spike-in mRNA
Map to Yeast Genome
Size-factor
RPF counts
DESeq2
Normalized RPFs
Input mRNA densities
REs
B
r =0.91
RLUC mAUG
FLUC mAUG
iAUGs of FLUC and RLUC
RPF -Ded1 #2 (log10)
RPF -Ded1 #1 (log10)
C
r = 0.96
RLUC mAUG
FLUC mAUG
iAUGs of FLUC and RLUC
RPF 100nM Ded1 #2 (log10)
RPF 100nM Ded1 #1 (log10)
D
r =0.90
RLUC mAUG
FLUC mAUG
iAUGs of FLUC and RLUC
RPF 500nM Ded1 #2 (log10)
RPF 500nM Ded1 #1 (log10)

Figure 1-S3

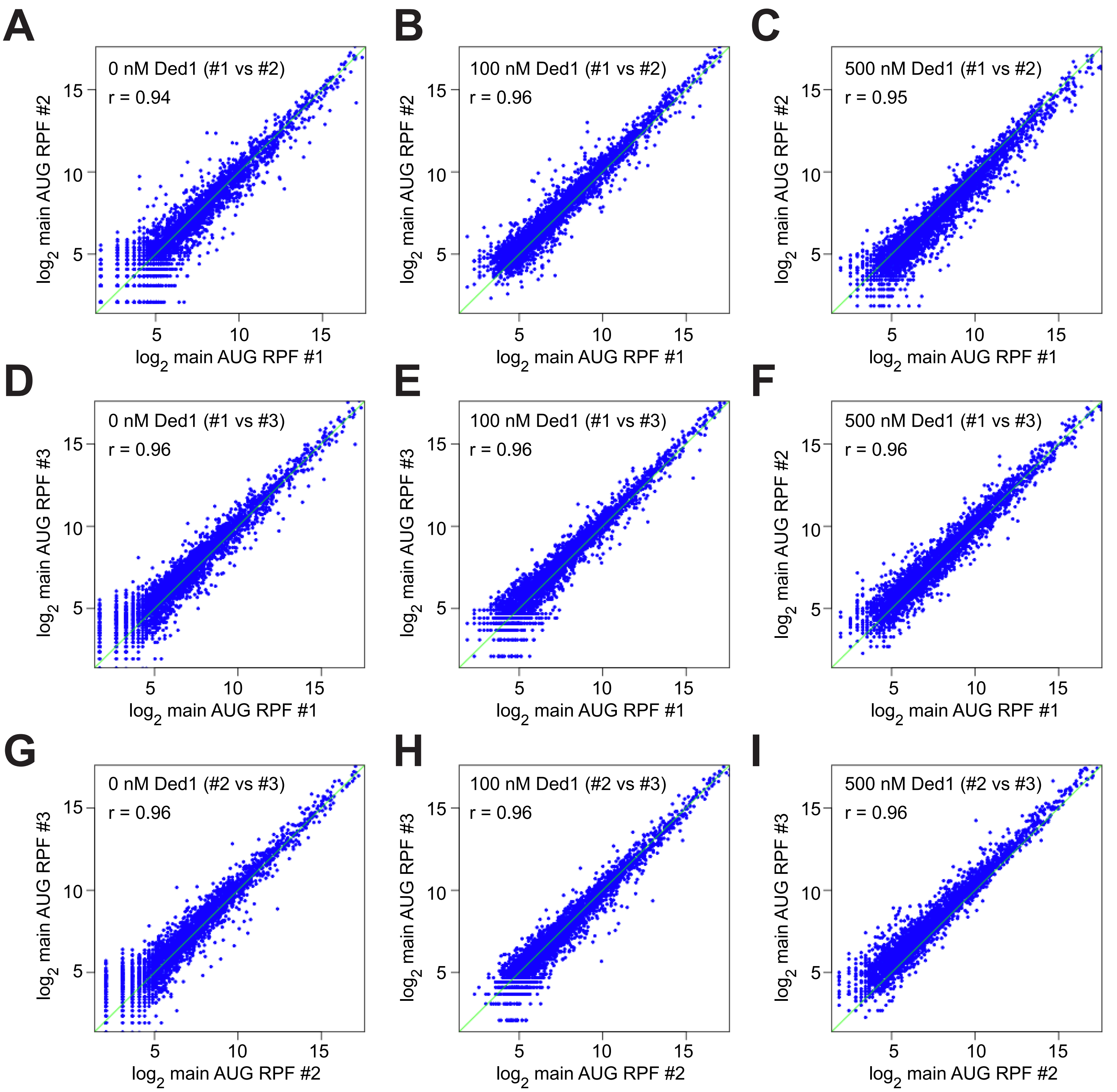

A
0 nM Ded1 (#1 vs #2)
r = 0.94
B
100 nM Ded1 (#1 vs #2)
r = 0.96
C
500 nM Ded1 (#1 vs #2)
r = 0.95
D
0 nM Ded1 (#1 vs #3)
r = 0.96
E
100 nM Ded1 (#1 vs #3)
r = 0.96
F
500 nM Ded1 (#1 vs #3)
r = 0.96
G
0 nM Ded1 (#2 vs #3)
r = 0.96
H
100 nM Ded1 (#2 vs #3)
r = 0.96
I
500 nM Ded1 (#2 vs #3)
r = 0.96
log2 main AUG RPF #1
log2 main AUG RPF #2
log2 main AUG RPF #3
5
10
15

Figure 3-S1

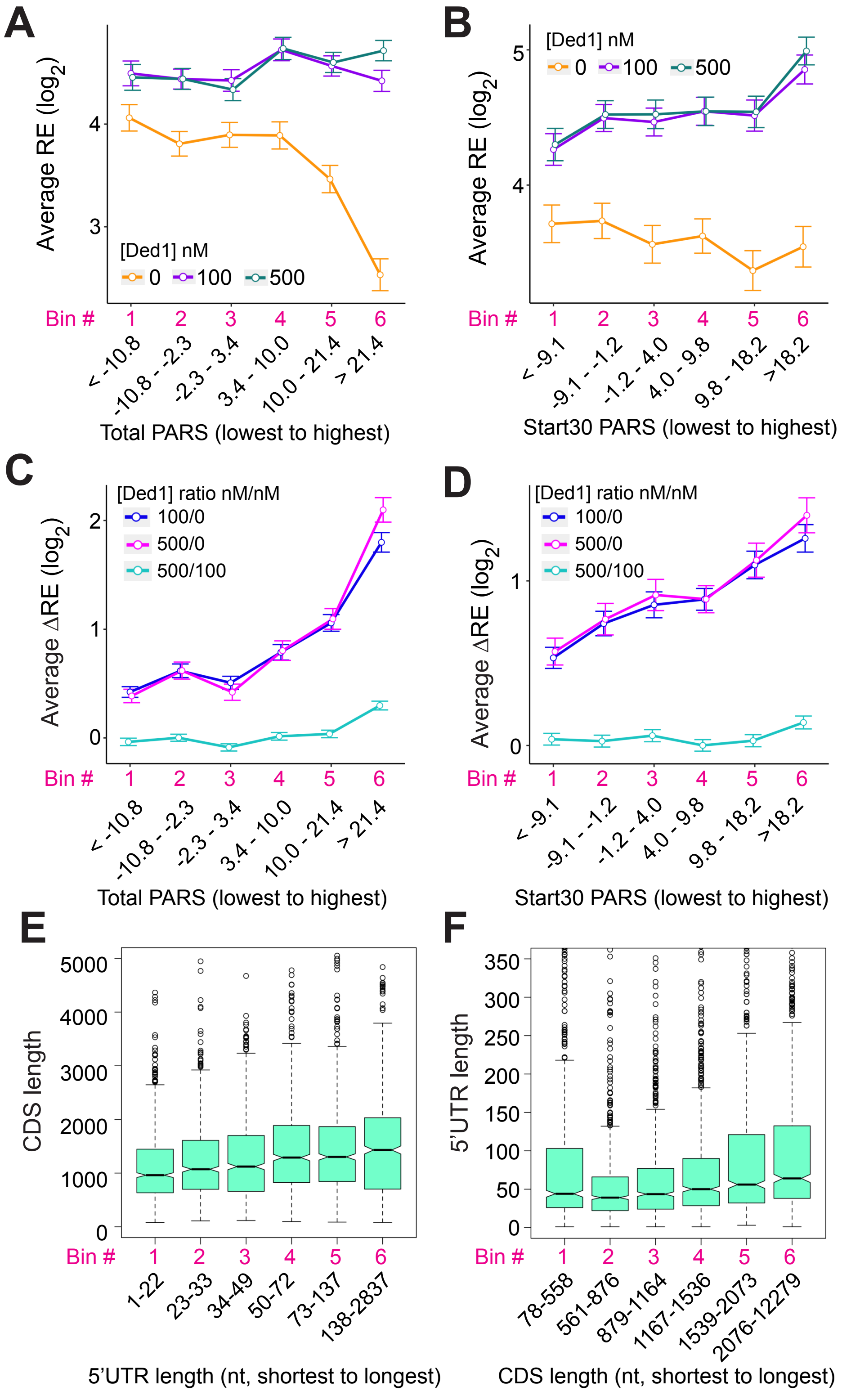
A
Average RE (log2)
[Ded1] nM
0
100
500
Bin #
1 2 3 4 5 6
< -10.8
-10.8 - -2.3
-2.3 - 3.4
3.4 - 10.0
10.0 - 21.4
> 21.4
Total PARS (lowest to highest)
B
Average RE (log2)
[Ded1] nM
0
100
500
Bin #
< -9.1
-9.1 - -1.2
-1.2 - 4.0
4.0 - 9.8
9.8 - 18.2
>18.2
Start30 PARS (lowest to highest)
C
Average ΔRE (log2)
[Ded1] ratio nM/nM
100/0
500/0
500/100
Total PARS (lowest to highest)
D
Average ΔRE (log2)
[Ded1] ratio nM/nM
100/0
500/0
500/100
Start30 PARS (lowest to highest)
E
CDS length
1-22
23-33
34-49
50-72
73-137
138-2837
5'UTR length (nt, shortest to longest)
F
5'UTR length
78-558
561-876
879-1164
1167-1536
1539-2073
2076-12279
CDS length (nt, shortest to longest)

Figure 3S-2

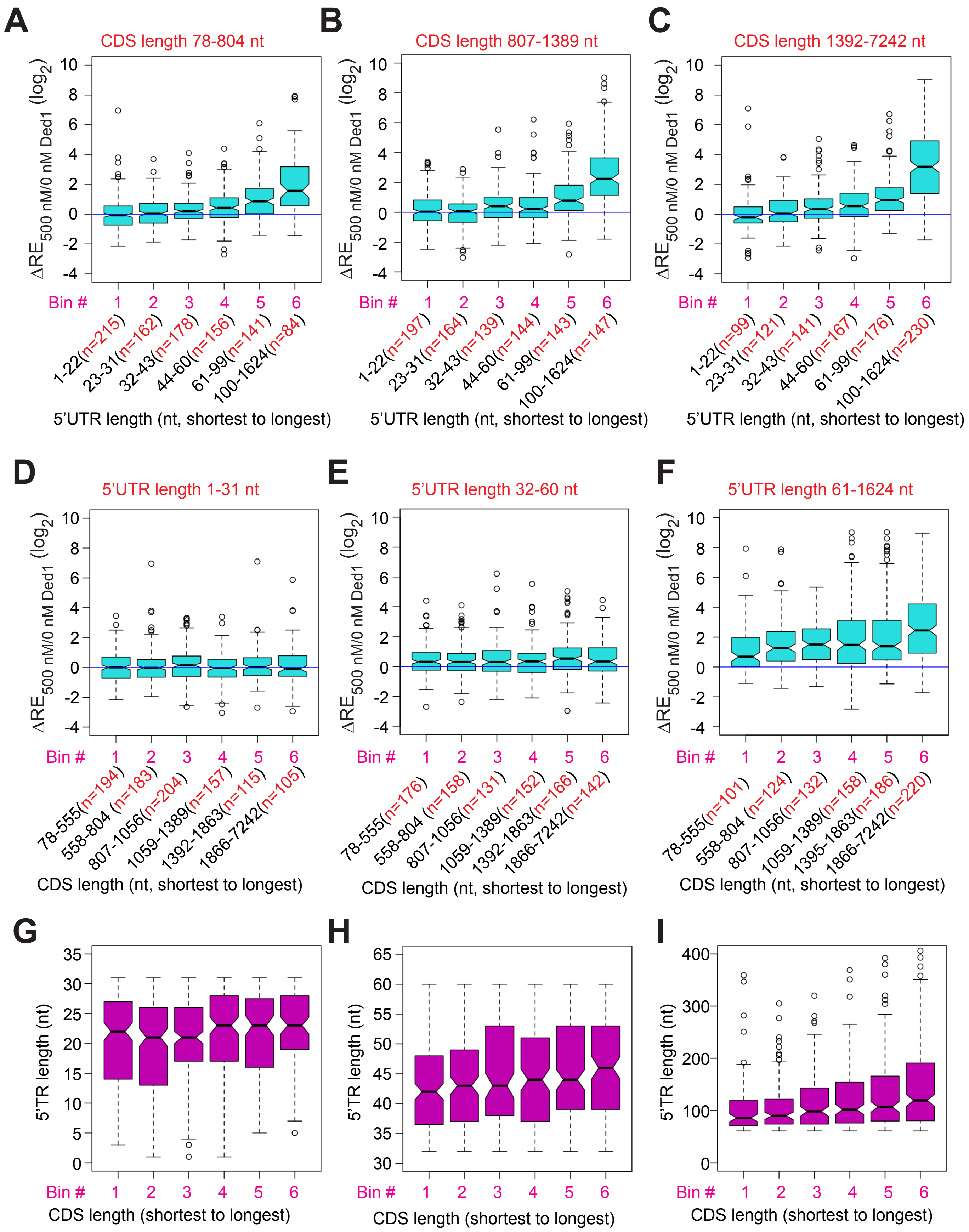
A
CDS length 78-804 nt
B
CDS length 807-1389 nt
C
CDS length 1392-7242 nt
ΔRE500 nM/0 nM Ded1 (log2)
Bin # 1 2 3 4 5 6
1-22(n=215) 23-31(n=162) 32-43(n=178) 44-60(n=156) 61-99(n=141) 100-1624(n=84)
1-22(n=197) 23-31(n=164) 32-43(n=139) 44-60(n=144) 61-99(n=143) 100-1624(n=147)
1-22(n=99) 23-31(n=121) 32-43(n=141) 44-60(n=167) 61-99(n=176) 100-1624(n=230)
5'UTR length (nt, shortest to longest)
D
5'UTR length 1-31 nt
E
5'UTR length 32-60 nt
F
5'UTR length 61-1624 nt
78-555(n=194) 558-804 (n=183) 807-1056(n=204) 1059-1389(n=157) 1392-1863(n=115) 1866-7242(n=105)
78-555(n=176) 558-804 (n=158) 807-1056(n=131) 1059-1389(n=152) 1392-1863(n=166) 1866-7242(n=142)
78-555(n=101) 558-804 (n=124) 807-1056(n=132) 1059-1389(n=158) 1395-1863(n=186) 1866-7242(n=220)
CDS length (nt, shortest to longest)
G
H
I
5'TR length (nt)
CDS length (shortest to longest)

Figure 3-S3

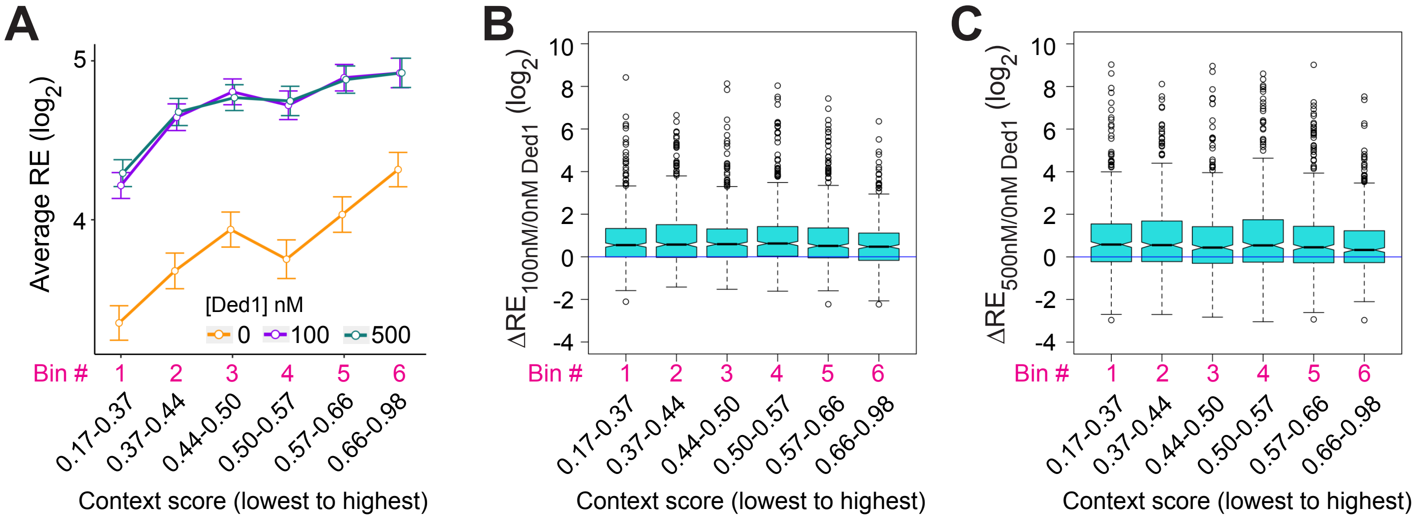

A
Average RE (log2)
5
4
[Ded1] nM
0
100
500
Bin #
1
2
3
4
5
6
0.17-0.37
0.37-0.44
0.44-0.50
0.50-0.57
0.57-0.66
0.66-0.98
Context score (lowest to highest)
B
ΔRE100nM/0nM Ded1 (log2)
10
8
6
4
2
0
-2
-4
Bin #
1
2
3
4
5
6
0.17-0.37
0.37-0.44
0.44-0.50
0.50-0.57
0.57-0.66
0.66-0.98
Context score (lowest to highest)
C
ΔRE500nM/0nM Ded1 (log2)
10
8
6
4
2
0
-2
-4
Bin #
1
2
3
4
5
6
0.17-0.37
0.37-0.44
0.44-0.50
0.50-0.57
0.57-0.66
0.66-0.98
Context score (lowest to highest)

Figure 5-S1

A

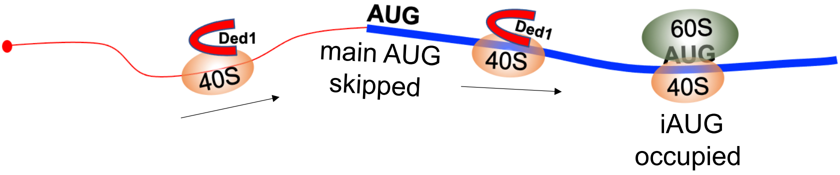

B

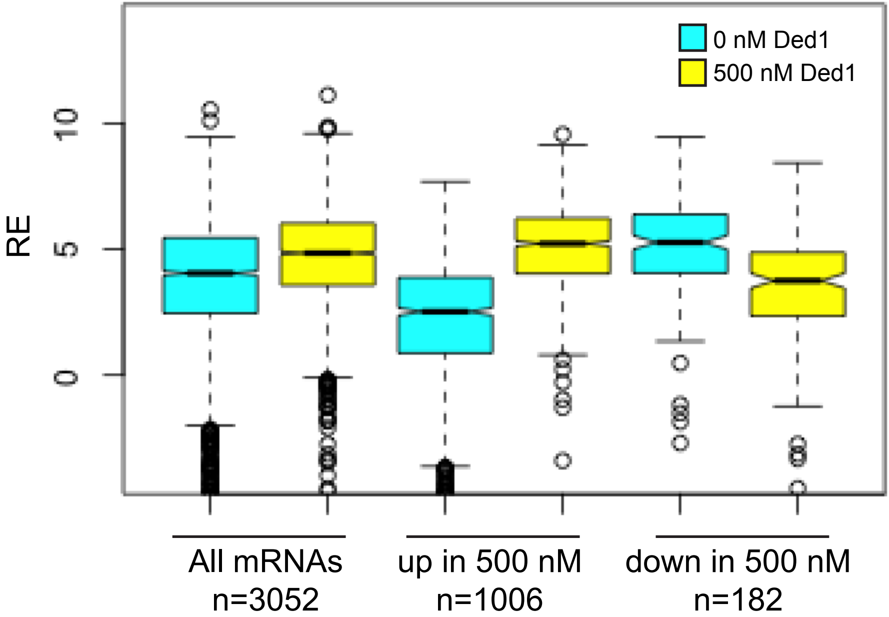

Figure 5-S2

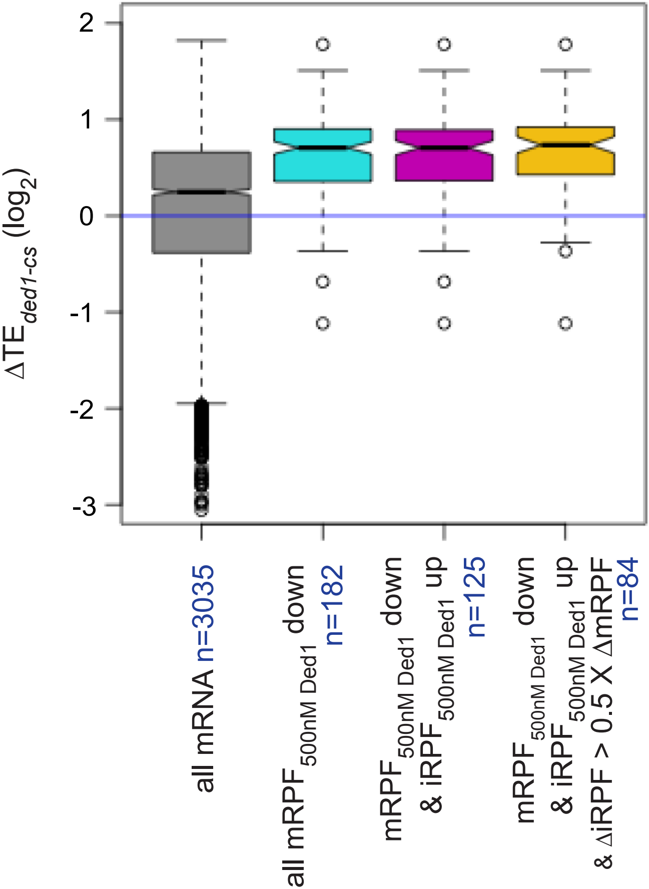

2
1
0
-1
-2
-3
ΔTE ded1-cs (log2)
all mRNA n=3035
all mRPF 500nM Ded1 down n=182
mRPF 500nM Ded1 down
& iRPF 500nM Ded1 up n=125
mRPF 500nM Ded1 down
& iRPF 500nM Ded1 up
& ΔiRPF > 0.5 X ΔmRPF n=84

Figure 6-S1

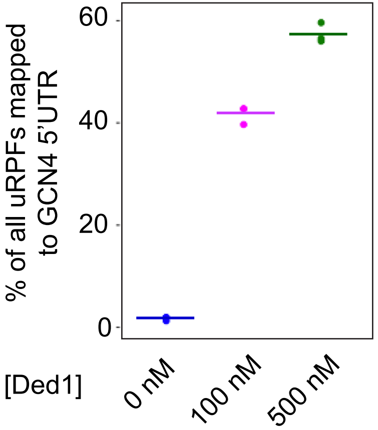

% of all uRPFs mapped
to GCN4 5'UTR
60
40
20
0
[Ded1]
0 nM
100 nM
500 nM
