# Supplementary figures and images for: Transcriptome-wide analysis of the function of Ded1 in translation preinitiation complex assembly in a reconstituted in vitro system
Source: bioRxiv. 2024 Feb 5:2023.10.16.562452. Originally published 2023 Oct 16. Preprint. [Version 2] doi: 10.1101/2023.10.16.562452 (PMC10659408; doi:10.1101/2023.10.16.562452)

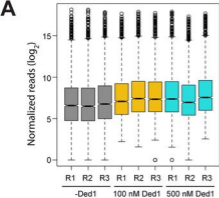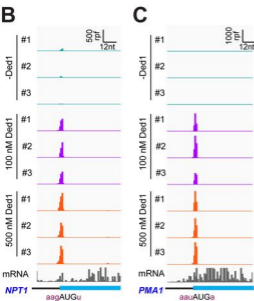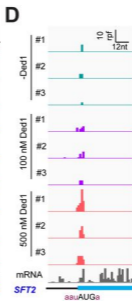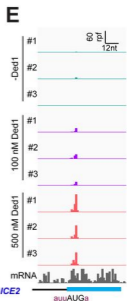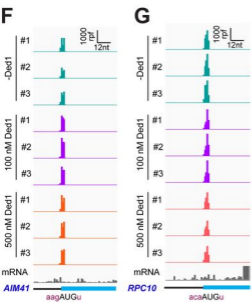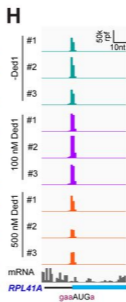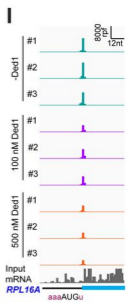

Supplement: Supplement 4 — Figure 1-S4. RPFs on main AUGs are consistent among replicates. (A) Box plot analysis of normalized RPF reads for 3052 mRNAs with > 90 total reads in 9 samples (each of 3 replicates for 0, 100 and 500 nM Ded1). (B-I) 48S PIC-protected fragments (RPFs) and input mRNA reads for 3 replicates of each of the 3 conditions (0, 100 and 500 nM Ded1) on 8 example mRNAs across 3' portions of the 5’UTR and 5' portions of the CDS regions flanking the main AUGs. RPF and nt scales are shown in the top right corner of each panel. The position of the main CDS and the −3 to −1 and +4 context nucleotides surrounding the main AUG are labeled as in Fig. 1E-F. mRNAs shown are (B) NPT1; (C) PMA1; (D) SFT2; (E) ICE2; (F) AIM41; (G) RCP10; (H) RPL41A; and (I) RPL16A. [file NIHPP2023.10.16.562452v2-supplement-4.pdf]
