# Supplementary material for: Transcriptome-wide analysis of the function of Ded1 in translation preinitiation complex assembly in a reconstituted in vitro system
Source: bioRxiv. 2023 Oct 16:2023.10.16.562452. Preprint. [Version 1] doi: 10.1101/2023.10.16.562452 (PMC10659408; doi:10.1101/2023.10.16.562452)
Supplement: Supplementary file 1 [file NIHPP2023.10.16.562452V1-supplement-1.pdf]

bioRxiv preprint doi: https://doi.org/10.1101/2023.10.16.562452; this version posted October 16, 2023. The copyright holder for this preprint (which was not certified by peer review) is the author/funder. This article is a US Government work. It is not subject to copyright under 17 USC 105 and is also made available for use under a CC0 license.

Table S1: Oligonucleotides used for Spike-in mRNA template amplification

**Primer name**	**Sequence (5' – 3')**
T7-FLUC	AAGGAATTCATCTTAACTTT**TAATACGACTCACTATAG**GGCAAACAAACAAACCAAAACCAAAACCACA***ATG***GAAGACGCCAAAAACATA
T7-RLUC	AAGGAATTCATCTTAACTTT**TAATACGACTCACTATAG**GGCAAACAAACAAACCAAAACCAAAACCACC***ATG***ACTTGCAAAGTTTATGAT
FLUC-R	GTCGACGAGGAATTCATTATCAGTGC
RLUC-R	GTCGACTTCTCCTTCTTCAGATTTGATC

Note: T7 promoter sequences are in boldface and underlined; mAUGs are in boldface and italicized

Figure 1-S1

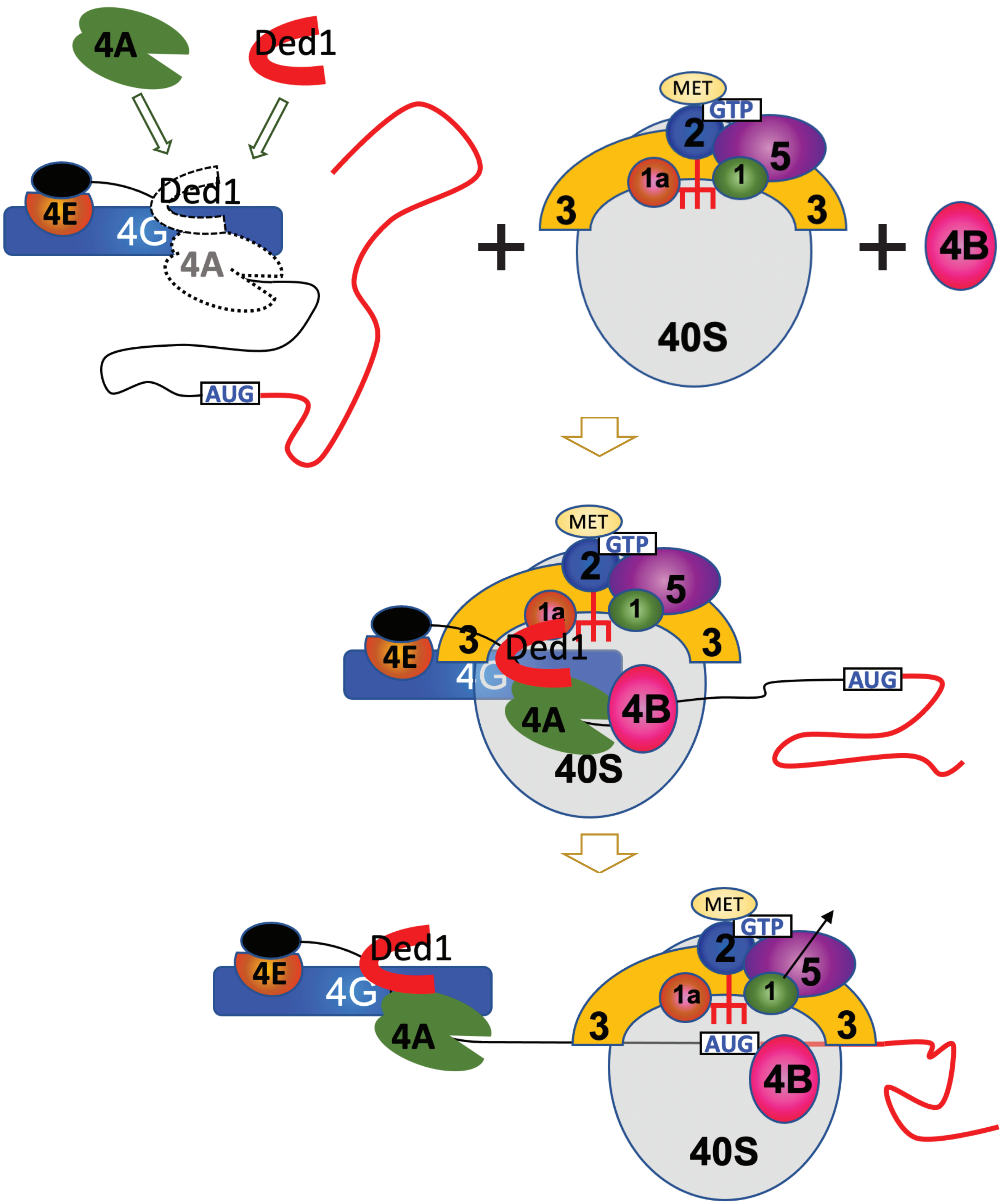

4A
Ded1
4E
4G
Ded1
4A
AUG
MET
GTP
2
5
1a
1
3
3
40S
4B
MET
GTP
2
5
1a
1
3
3
4E
Ded1
4G
4A
4B
40S
AUG
MET
GTP
2
5
4E
Ded1
4G
1a
1
3
3
4A
AUG
4B
40S

Figure 1-S1. A schematic diagram of mRNA recruitment in translation initiation. The cap-binding protein complex eIF4F, consisting of eIF4E, eIF4G and eIF4A subunits attaches to the cap at the mRNA 5' end. The 43S PIC, which includes the 40S ribosomal subunit, eIFs 1, 1A, 3 and 5 and the eIF2•GTP•Met-tRNA$_i$ ternary complex, binds to the 5' end of mRNA with the aid of the eIF4F complex and eIF4B and scans downstream in search of the start codon. A stable 48S-PIC is formed when a start codon is recognized.

Figure 1-S2

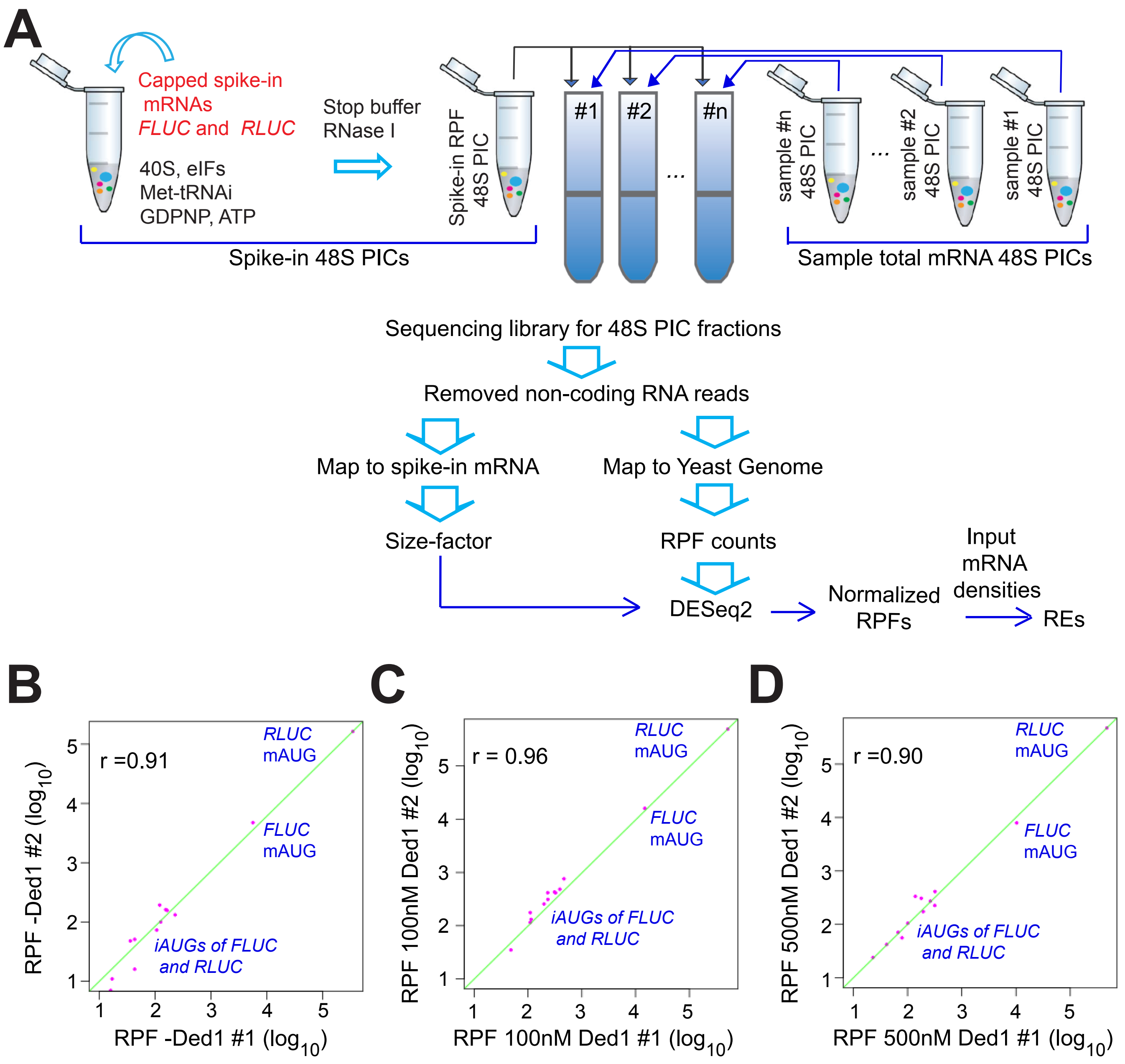

A
Capped spike-in mRNAs
FLUC and RLUC
40S, eIFs
Met-tRNAi
GDPNP, ATP
Stop buffer
RNase I
Spike-in RPF 48S PIC
#1
#2
#n
sample #n 48S PIC
sample #2 48S PIC
sample #1 48S PIC
Spike-in 48S PICs
Sample total mRNA 48S PICs
Sequencing library for 48S PIC fractions
Removed non-coding RNA reads
Map to spike-in mRNA
Map to Yeast Genome
Size-factor
RPF counts
DESeq2
Normalized RPFs
Input mRNA densities
REs
B
r =0.91
RLUC mAUG
FLUC mAUG
iAUGs of FLUC and RLUC
RPF -Ded1 #2 (log10)
RPF -Ded1 #1 (log10)
C
r = 0.96
RLUC mAUG
FLUC mAUG
iAUGs of FLUC and RLUC
RPF 100nM Ded1 #2 (log10)
RPF 100nM Ded1 #1 (log10)
D
r =0.90
RLUC mAUG
FLUC mAUG
iAUGs of FLUC and RLUC
RPF 500nM Ded1 #2 (log10)
RPF 500nM Ded1 #1 (log10)

Figure 1-S2. Internal spike-in normalization controls using preformed 48S PICs on non-native mRNAs. **(A)** Steps in applying 48S-PIC spike-in controls for data normalization. Firefly and Renilla luciferase mRNAs (*FLUC* and *RLUC)* were in vitro transcribed from PCR-amplified gene templates containing T7 promoters and enzymatically capped using previously described protocols (Acker et al. 2007). The 48S PICs formed on the spike-in mRNAs were prepared separately from the testing samples in a recruitment reaction followed by RNase I treatment to digest unprotected mRNA. A constant amount of spike-in 48S PICs were resolved by sedimentation through the same sucrose gradients containing the experimental samples. The RPFs from these spike-in PICs served as internal controls for losses occurring during library construction and allowed data normalization across experimental samples. For deep sequencing data processing, non-coding RNA reads were removed and the remaining RPFs were aligned to spike-in mRNA sequences for calculating sample size factors and also aligned to the yeast genome to produce RPF counts on all yeast mRNAs. The RPF counts for all mRNAs in the genome were applied to DESeq2 using sample size factors calculated from the geometric means of spike-in mRNA RPFs mapped to *FLUC*/*RLUC* main AUGs and a number of internal AUGs. Recruitment efficiency (RE) values were calculated by normalizing the size-factor corrected RPFs to input mRNA densities. **(B-D)** Scatterplots of *FLUC* and *RLUC* RPF reads at the main and internal AUGs, in the first two of three replicate samples for each condition. The green diagonal line is for 1:1 ratio and the Spearman correlation coefficients (r) were calculated using "cor.test" function in R using option "method = spearman."

Figure 1-S3

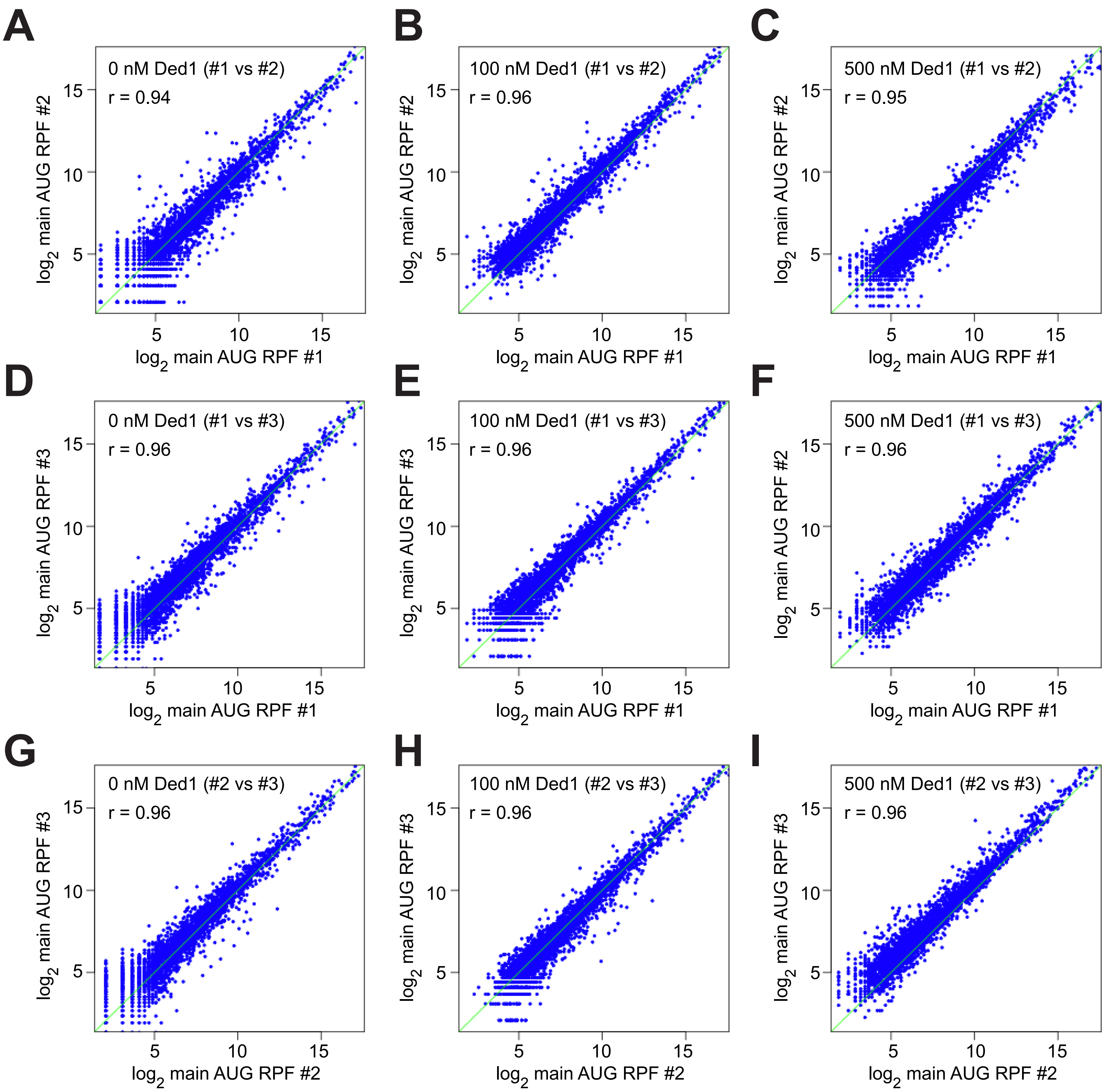

A
0 nM Ded1 (#1 vs #2)
r = 0.94
B
100 nM Ded1 (#1 vs #2)
r = 0.96
C
500 nM Ded1 (#1 vs #2)
r = 0.95
D
0 nM Ded1 (#1 vs #3)
r = 0.96
E
100 nM Ded1 (#1 vs #3)
r = 0.96
F
500 nM Ded1 (#1 vs #3)
r = 0.96
G
0 nM Ded1 (#2 vs #3)
r = 0.96
H
100 nM Ded1 (#2 vs #3)
r = 0.96
I
500 nM Ded1 (#2 vs #3)
r = 0.96
log2 main AUG RPF #1
log2 main AUG RPF #2
log2 main AUG RPF #3
5
10
15

Figure 1-S3. Transcriptome-wide reproducibility of RPFs among replicates
(A-I) Scatterplots of log2 main AUG ribosome occupancies (normalized by the geometric means of RPF reads on *FLUC* and *RLUC* spike-in mRNAs) for comparing each of two of the three replicates in different conditions, 0 nM Ded1 (A, D and G), 100 nM Ded1 (B, E and H) and 500 nM Ded1 (C, F and I) for all mRNAs with ⩾ 90 total RPF reads in the 9 samples combined. Spearman correlation coefficients (r) were calculated as in Fig. 1-S2B-D.

Figure 1-S4

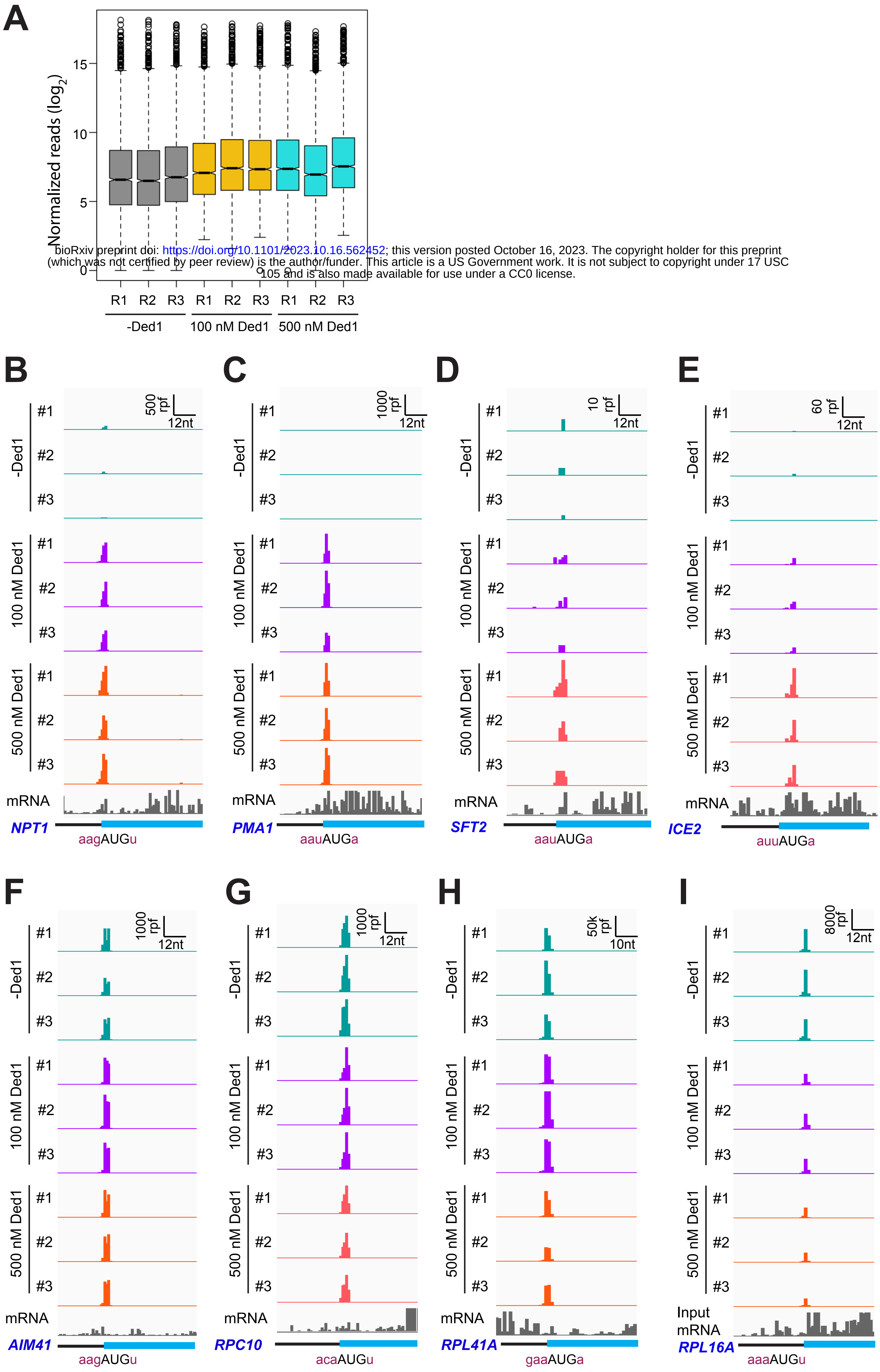

bioRxiv preprint doi: https://doi.org/10.1101/2023.10.16.562452; this version posted October 16, 2023. The copyright holder for this preprint (which was not certified by peer review) is the author/funder. This article is a US Government work. It is not subject to copyright under 17 USC 105 and is also made available for use under a CC0 license.

Figure 1-S4. RPFs on main AUGs are consistent among replicates.
(A) Box plot analysis of normalized RPF reads for 3052 mRNAs with > 90 total reads in 9 samples (each of 3 replicates for 0, 100 and 500 nM Ded1). **(B-I)** 48S PIC-protected fragments (RPFs) and input mRNA reads for 3 replicates of each of the 3 conditions (0, 100 and 500 nM Ded1) on 8 example mRNAs across 3' portions of the 5'UTR and 5' portions of the CDS regions flanking the main AUGs. RPF and nt scales are shown in the top right corner of each panel. The position of the main CDS and the −3 to −1 and +4 context nucleotides surrounding the main AUG are labeled as in Fig. 1E-F. mRNAs shown are (B) *NPT1*; (C) *PMA1*; (D) *SFT2*; (E) *ICE2*; (F) *AIM41*; (G) *RCP10*; (H) *RPL41A;* and (I) *RPL16A*.

Figure 3-S1

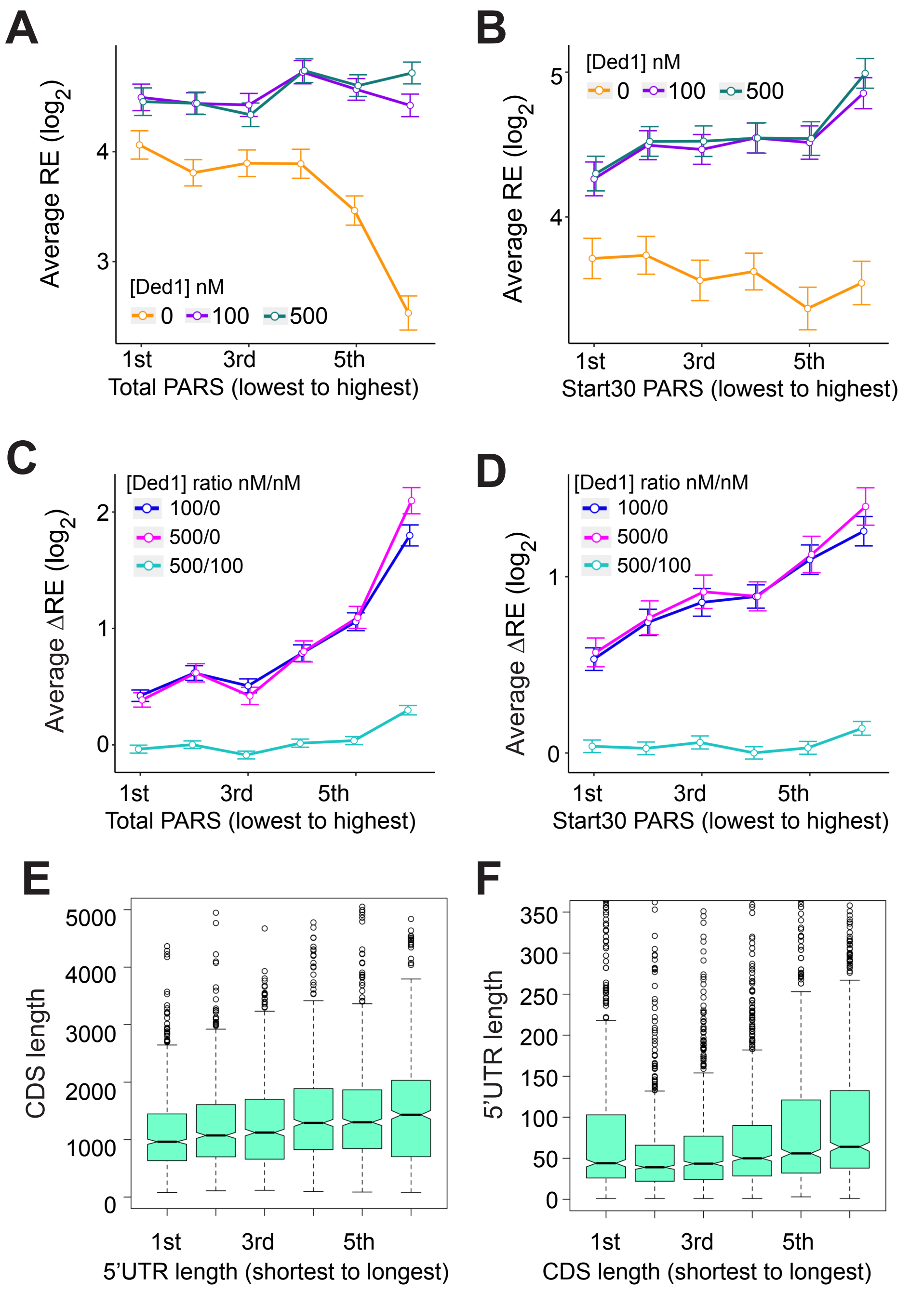

A
Average RE (log2)
4
3
[Ded1] nM
0
100
500
1st
3rd
5th
Total PARS (lowest to highest)
B
Average RE (log2)
5
4
[Ded1] nM
0
100
500
1st
3rd
5th
Start30 PARS (lowest to highest)
C
Average ΔRE (log2)
2
1
0
[Ded1] ratio nM/nM
100/0
500/0
500/100
1st
3rd
5th
Total PARS (lowest to highest)
D
Average ΔRE (log2)
1
0
[Ded1] ratio nM/nM
100/0
500/0
500/100
1st
3rd
5th
Start30 PARS (lowest to highest)
E
CDS length
5000
4000
3000
2000
1000
0
1st
3rd
5th
5'UTR length (shortest to longest)
F
5'UTR length
350
300
250
200
150
100
50
0
1st
3rd
5th
CDS length (shortest to longest)

Figure 3-S1. Ded1 preferentially stimulates 48S PIC formation on structured mRNAs. (A, B) Line plot analysis of log2 average REs at 0, 100 and 500 nM Ded1for mRNAs binned according to total PARS scores (A) or the sum of PARS scores of the 30 nucleotides surrounding the start codon (for mRNAs with a 5'UTR of ⩾15 nt) (Start30 PARS, B). **(C, D)** Line plot analysis of log2 average ΔREs for 100/0 nM, 500/0 nM and 500/100 nM Ded1 for the 2035 mRNAs observed in RecSeq that have annotated PARS scores binned according to Total PARS (C) or Start30 PARS (D) scores from the lowest to highest. **(E-F)** Box plots comparing 5'UTR or CDS lengths for the 4694 yeast mRNAs that have annotated 5'UTRs (Pelechano et al. 2013) for CDS lengths plotted for mRNAs binned by 5'UTR length from the shortest to the longest (E), or 5'UTR lengths plotted for mRNAs binned by CDS lengths from shortest to longest (F). All bins contain equal numbers of mRNAs.

Figure 3-S2

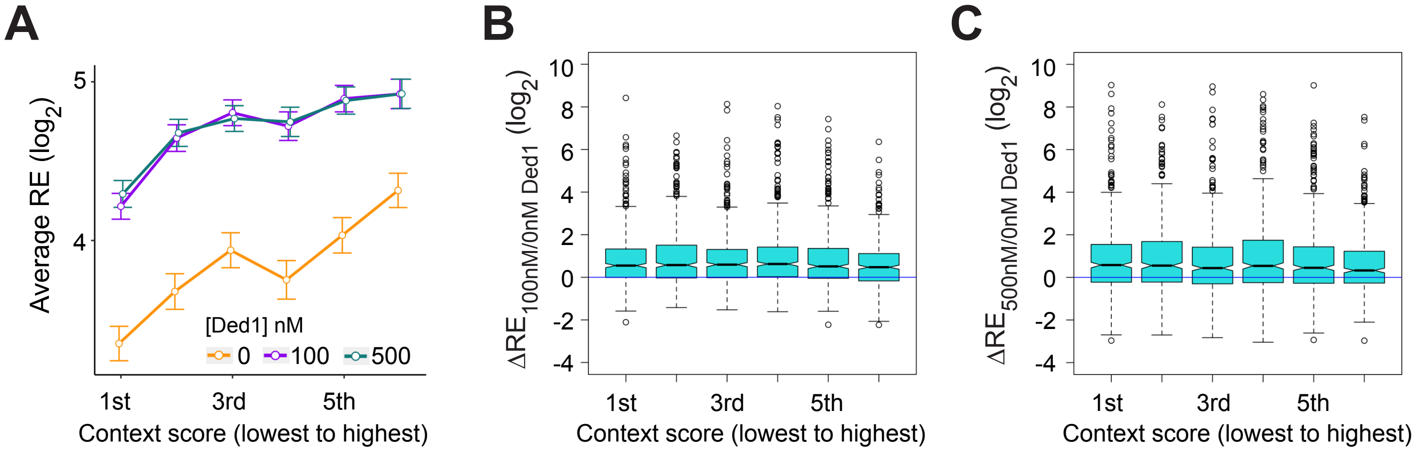
A
B
C
Average RE ($\log_2$)
[Ded1] nM
0
100
500
5
4
$\Delta RE_{100nM/0nM\ Ded1}$ ($\log_2$)
$\Delta RE_{500nM/0nM\ Ded1}$ ($\log_2$)
10
8
6
4
2
0
-2
-4
1st
3rd
5th
Context score (lowest to highest)

Figure 3-S2. Ded1 has little effect on discriminating main AUG context scores. (A) Line plot analysis of average REs for the 2768 observed in RecSeq that have annotated 5'UTR lengths >5 nt, divided into six equal-sized bins according to context scores from lowest to the highest at 0, 100 and 500 nM Ded1. **(B-C)** Box-plot analyses of $\Delta RE_{100/0nM\ Ded1}$ (B) and $\Delta RE_{500/0nM\ Ded1}$ (C) values for the same mRNA bins as in (A).

Figure 5-S1

A

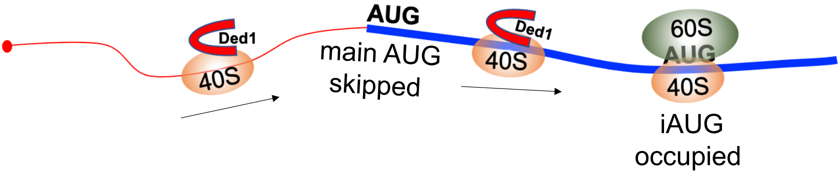

B

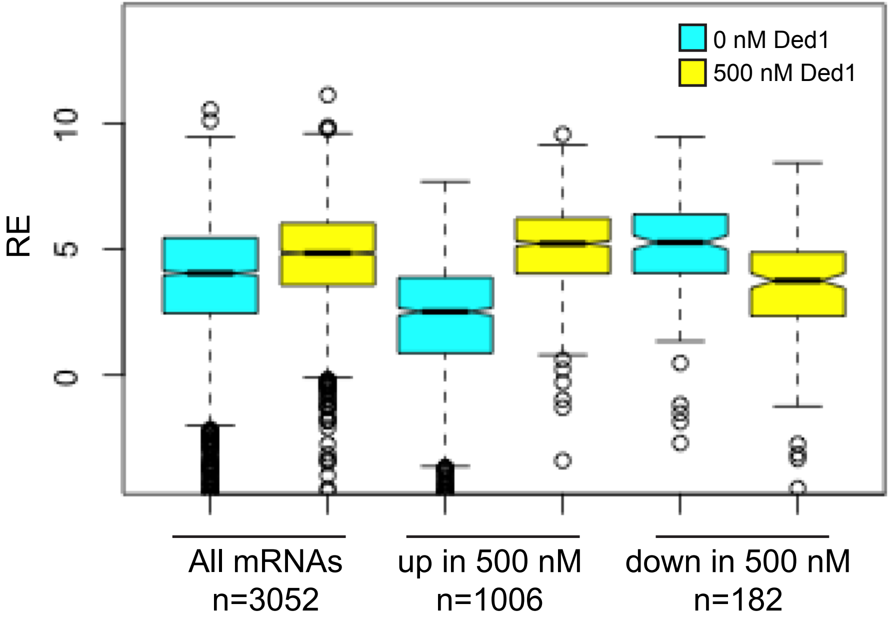

Figure 5-S1. Supporting evidence that Ded1 promotes leaky scanning of the main start codon. (A) Schematic of a 43S PIC leaky scanning through the main AUG and assembling at an internal AUG in the presence of Ded1. **(B)** Evidence that mRNAs whose recruitment is inhibited by 500 nM Ded1 in Rec-Seq assays generally assemble 48S PICs very efficiently without Ded1. Boxplot analysis of REs of groups of mRNAs at 0 nM (cyan) or 500 nM (yellow) Ded1 for all 3052 mRNAs observed in Rec-Seq, the 1006 mRNAs with significantly increased mRPFs at 500 nM Ded1 versus no Ded1 (Fig. 2B, red dots); and the 182 mRNAs with significantly decreased mRPFs with 500 nM Ded1 versus no Ded1 (Fig. 2B, blue dots).

Figure 5-S2

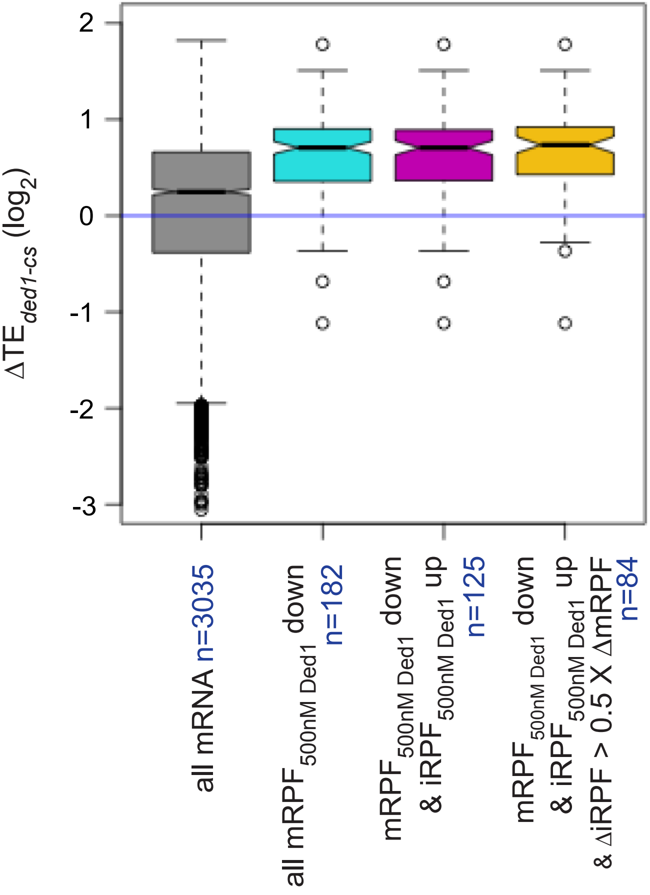

2
1
0
-1
-2
-3
ΔTE ded1-cs (log2)
all mRNA n=3035
all mRPF 500nM Ded1 down n=182
mRPF 500nM Ded1 down & iRPF 500nM Ded1 up n=125
mRPF 500nM Ded1 down & iRPF 500nM Ded1 up & ΔiRPF > 0.5 X ΔmRPF n=84

Figure 5-S2. mRNAs with reduced RE at 500 nM Ded1 versus no Ded1 in Rec-Seq assays tend to show increased relative TE in the *ded1-cs* mutant versus WT in ribosome profiling experiments. Boxplot analysis of $\Delta TE_{ded1\text{-}cs}$ for the groups of mRNAs described in Figure 5L-M: The 182 mRNAs with mRPFs significantly reduced at 500 nM Ded1 versus no Ded1 (cyan); the 125 mRNAs with significantly decreased mRPFs and increased iRPFs with 500 nM Ded1 versus no Ded1 (magenta); the 84 mRNAs from the previous set that had an increase in iRPFs that was at least 50% the of the decrease in mRPFs (yellow circles in Fig. 5M; orange).

Figure 6-S1

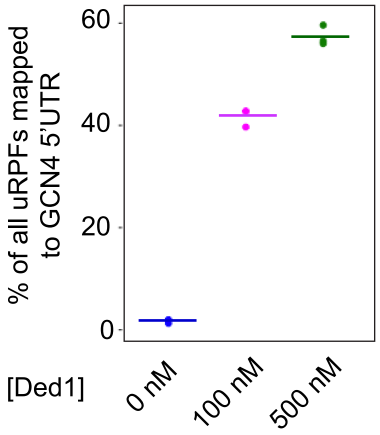

% of all uRPFs mapped to GCN4 5'UTR
60
40
20
0
[Ded1]
0 nM
100 nM
500 nM

Figure 6-S1. Approximately half of all uRPFs map to the *GCN4* 5'UTR. Percentages of all uRPFs mapped to the *GCN4* 5'UTR for each of the three replicates with 0 (blue), 100 (magenta), or 500 (green) nM Ded1 concentrations. Colored bars indicate the means of the replicates.
